# Abrupt Subsidence of Seasonal Influenza after COVID-19 Outbreak, Hong Kong, China

**DOI:** 10.3201/eid2611.200861

**Published:** 2020-11

**Authors:** Ngai-Sze Wong, Chi-Chiu Leung, Shui-Shan Lee

**Affiliations:** The Chinese University of Hong Kong, Hong Kong, China (N.-S. Wong, S.-S. Lee);; Hong Kong Tuberculosis, Chest and Heart Diseases Association, Hong Kong (C.-C. Leung)

**Keywords:** coronavirus, viruses, severe acute respiratory syndrome coronavirus 2, SARS-CoV-2, coronavirus disease, respiratory infections, influenza, seasonal influenza, influenza virus, subsistence, zoonoses, Hong Kong, China

## Abstract

The onset of the 2019–20 winter influenza season in Hong Kong coincided with the emergence of the coronavirus disease epidemic in neighboring mainland China. After widespread adoption of large-scale social distancing interventions in response to the impending coronavirus disease outbreak, the influenza season ended abruptly with a decrease to a low trough.

Seasonality is a distinctive feature of influenza epidemics, the pattern of which is shaped by host, virus, and environmental factors (*1*). An influenza season typically lasts for an average of 13 weeks (https://www.cdc.gov/flu/pastseasons/1112season.htm) in the United States, and seasons might range from 6.5 to 21.4 weeks, as reported in a study in Europe (*2*). Seasonal influenza in Hong Kong, China, which is located in the Northern Hemisphere, is characterized by dual peaks: a winter peak frequently occurring within the first 2 months of the year, followed by a less prominent summer peak in August or September of the same year. Toward the end of December 2019, emergence of severe acute respiratory syndrome coronavirus 2 (SARS-CoV-2) was reported in Wuhan (*3*), the timing of which coincided with the onset of the 2019–20 winter influenza season in Hong Kong.

Using surveillance data accessed from the Hong Kong Government Centre for Health Protection (https://www.chp.gov.hk/en/resources/29/304.html), we assessed the epidemic pattern of the concurrent winter influenza season and explored its temporal relationship with prevailing interventions. We found that the 2019–20 winter season had its onset in late November, reaching its peak 7 weeks later, and decreasing precipitously to a trough after 1 month (Figure).

**Figure:**
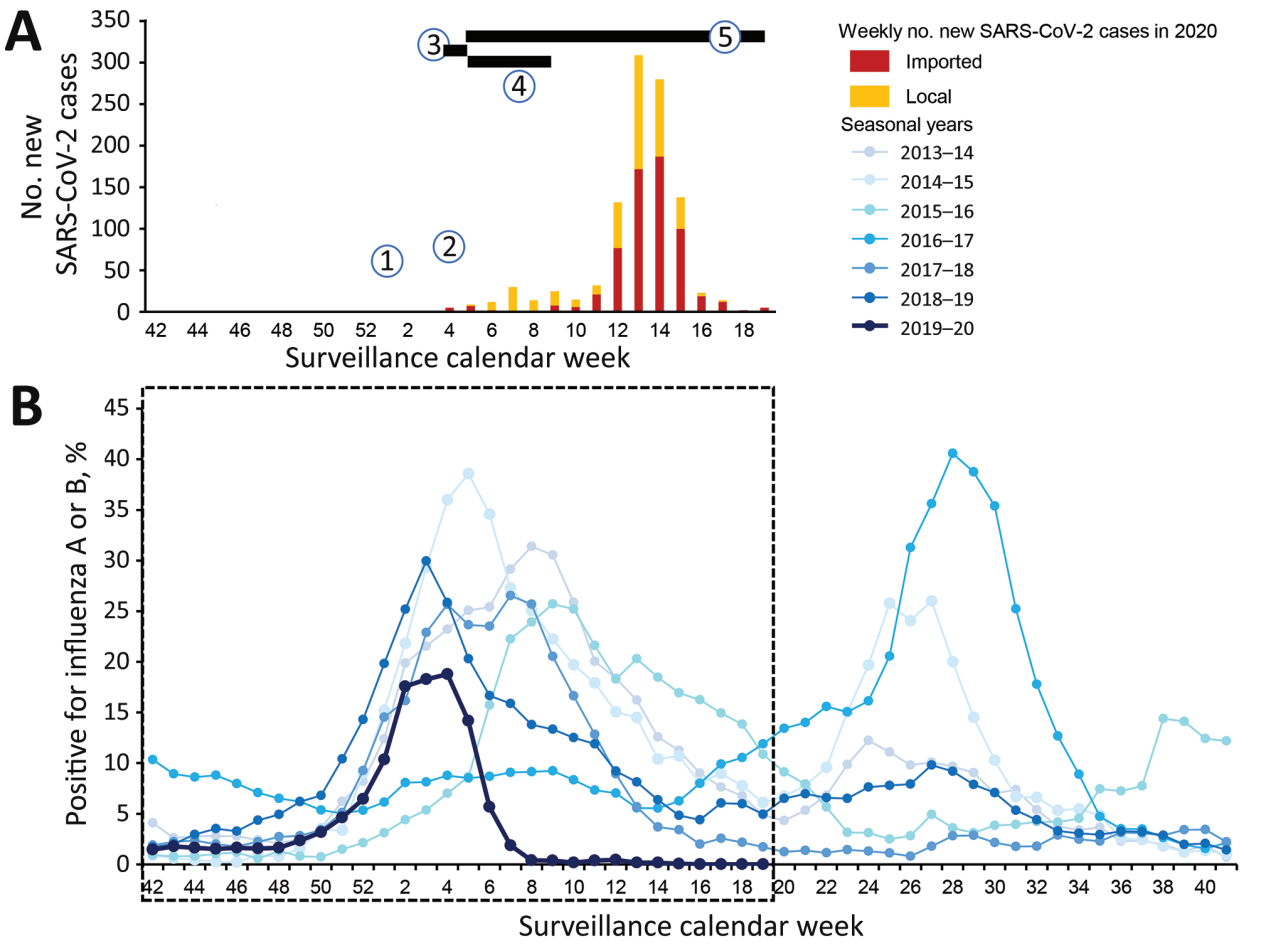
Epidemic curve showing number of reported SARS-CoV-2 cases (A) in Hong Kong, China, and abrupt subsidence during the corresponding 2019–20 winter influenza season (B) compared with 6 preceding years between 2013 and 2019, as derived from the percentage of respiratory specimens tested that were positive for influenza A or B viruses. Onset of winter influenza season is defined as the first week that had an increase in percentage of respiratory samples tested that were positive for influenza A or B viruses, followed by a consecutive increase for >4 weeks. End of the season is defined as the last week that had a decrease of the same percentage, followed by a consecutive decrease for >2 weeks, compared with the previous week. SARS-CoV-2 timelines: 1, on December 30, 2019, the Wuhan Municipal Health Committee issued an urgent notice on treatment for pneumonia of unknown cause; 2, on January 25, 2020, the World Health Organization declared a Public Health Emergency of International Concern; 3, during January 25–28, 2020, the Lunar New Year public holiday occurred; 4, during January 29–March 1, 2020, civil servants made special work arrangements; 5, during January 29–May 26, 2020, schools were closed. SARS-CoV-2, severe acute respiratory syndrome coronavirus 2.

Compared with the winter seasons in the preceding 6 years, the 2019–20 winter season was shorter (13 weeks vs. median 22 weeks, interquartile range [IQR] 14–25 weeks), had a relatively small peak (18.8% vs. median 26.5%, IQR 18.8%–31.4%), and decreased to a much lower postseason trough on its subsidence (0.4% vs median 4.3%, IQR 0.8%–5.5%) (Table). The decrease was abrupt and had a peak-to-trough median interval of 6 weeks versus 14 weeks (IQR 6–17 weeks). The median weekly number of respiratory specimens tested during the 2019–20 winter season was also higher (5,711 vs. 4,104, IQR 3,640–5,711).

**Table:** Characteristics of winter influenza seasons, Hong Kong, China, 2013–14 through 2019–20*

Seasonal year	Onset week	Duration, weeks	Peak level, %	Postseason trough, %	Peak to trough, wk	Weekly no. tests, median (IQR)
2013–14	49	22	31.4	4.3	13	2,423 (2,191–2,588)
2014–15	49	21	38.6	6.1	15	3,640 (3,029–4,230)
2015–16	51	25	25.7	2.5	17	4,017 (3,292–4,537)
2016–17	52	14	9.2	5.5	6	4.104 (3,890–4,216)
2017–18	47	30	26.5	0.8	20	4,426 (4,100–6,335)
2018–19	47	23	29.9	4.4	14	6,073 (5,439–6,475)
2019–20	48	13	18.8	0.4	6	5,711 (5,327–6,318)

*IQR, interquartile range.

This pattern contrasted with that for the United States, in which increased influenza-like illness activity was first noted in November 2019 (*4*). Influenza activity remained increased for >4 months, through March 2020, without any sign of abrupt subsidence in the United States. Influenza A(H1N1)pdm09 was the dominant virus for the 2019–20 season in Hong Kong (https://www.chp.gov.hk/files/pdf/fluexpress_week2_16_1_2020_eng.pdf) and the United States (*4*). However, the second most common virus was H3N2 subtype virus in Hong Kong but influenza B/Victoria virus in United States.

To explain the unique epidemic pattern of the 2019–20 winter influenza season, we matched the epidemic curve with the corresponding milestones related to the emergence of SARS-CoV-2 in Hong Kong (Figure). From the last week of January 2020 (influenza surveillance calendar week 4), the government had implemented school closure and mandated work-from-home arrangements for civil servants immediately after the Lunar New Year Holiday. This implementation was followed by closure of most borders with mainland China during the first week of February 2020. The effect was dramatic because vacating of workplaces affected not only the government staff force of 170,000 but also employees of statutory bodies, nongovernment organizations, and major businesses. Universities, secondary and primary schools, and kindergartens were closed and were not expected to reopen by late April. Mass gatherings, including the Lunar New Year celebration, and sports events were cancelled; church services had largely ceased; and the overcrowded public transportation system had eased substantially. Another common sight was mass masking, which has occurred as an adopted precaution voluntarily by most local citizens as a result of panic and as advocated by the medical profession (*5*). The phenomenon might have played a supplemental role in reducing the opportunity of virus exposure, although its precise effect has yet to be confirmed.

The early plateauing and abrupt decrease of influenza activities toward the end of January 2020 was temporally correlated with the extended social distancing effects of the government’s SARS-CoV-2 mitigation strategy. Overall, the abrupt subsidence of influenza activities and suppressed postseason trough were unique characteristics of the 2019–20 influenza season, differing substantially from the continually high peak of the parallel season in the United States.

We acknowledge that our assessment could be limited by the application of retrospective and descriptive methods involving analyses of publicly available surveillance data. It is possible that the temporal relationship between the seasonal influenza pattern and social distancing strategy implementation had occurred coincidentally by chance because heterogeneity of influenza seasons is a well-known phenomenon. Previous research suggested that despite the marked fluctuations of peak amplitudes and peak times, epidemic duration is often conserved (*2*). However, occurrence of a deformed seasonal pattern in the setting of the outbreak of infection with SARS-CoV-2 served as a natural experiment for supporting the evaluation of the impacts of social distancing in mitigating influenza virus transmission (*6*,*7*).
